# Aneuploidy and Ethanol Tolerance in *Saccharomyces cerevisiae*

**DOI:** 10.3389/fgene.2019.00082

**Published:** 2019-02-12

**Authors:** Miguel Morard, Laura G. Macías, Ana C. Adam, María Lairón-Peris, Roberto Pérez-Torrado, Christina Toft, Eladio Barrio

**Affiliations:** ^1^Departament de Genètica, Universitat de València, Valencia, Spain; ^2^Departamento de Biotecnología, Instituto de Agroquímica y Tecnología de los Alimentos (IATA), CSIC, Valencia, Spain

**Keywords:** *Saccharomyces cerevisiae*, wine yeasts, chromosome III, aneuploidy, comparative genomics, ethanol tolerance

## Abstract

Response to environmental stresses is a key factor for microbial organism growth. One of the major stresses for yeasts in fermentative environments is ethanol. *Saccharomyces cerevisiae* is the most tolerant species in its genus, but intraspecific ethanol-tolerance variation exists. Although, much effort has been done in the last years to discover evolutionary paths to improve ethanol tolerance, this phenotype is still hardly understood. Here, we selected five strains with different ethanol tolerances, and used comparative genomics to determine the main factors that can explain these phenotypic differences. Surprisingly, the main genomic feature, shared only by the highest ethanol-tolerant strains, was a polysomic chromosome III. Transcriptomic data point out that chromosome III is important for the ethanol stress response, and this aneuploidy can be an advantage to respond rapidly to ethanol stress. We found that chromosome III copy numbers also explain differences in other strains. We show that removing the extra chromosome III copy in an ethanol-tolerant strain, returning to euploidy, strongly compromises its tolerance. Chromosome III aneuploidy appears frequently in ethanol-tolerance evolution experiments, and here, we show that aneuploidy is also used by natural strains to enhance their ethanol tolerance.

## Introduction

The yeast *Saccharomyces cerevisiae* is among the most beneficial microorganisms for humans, especially industrial strains involved in the production of fermented products, such as bread, beer or wine. *S. cerevisiae*, as well as other *Saccharomyces* species, are characterized by their ability to ferment simple sugars into ethanol, even when oxygen is available for aerobic respiration (Crabtree effect), due to an overflow in the glycolysis pathway (Hagman and Piškur, 2015). Although, alcohol fermentation is energetically less efficient than respiration, it provides a selective advantage to these yeasts to out-compete other microorganisms. This way, sugar resources are consumed faster and the ethanol produced during fermentation, as well as high levels of heat and CO_2_, can be harmful or less tolerated by their competitors. Once competitors are overcome, *Saccharomyces* yeasts can use the accumulated ethanol as a substrate for aerobic respiration in the presence of oxygen. This ecological strategy was named (ethanol) “make-accumulate-consume" (Thomson et al., 2005; Piškur et al., 2006).

With the advent of the human hunter-gatherer societies, *S. cerevisiae*, due to its fermentative capabilities, successfully occupied a new ecological niche in the crushed grape berries, collected by humans to produce the first fermented beverages. With agriculture, Neolithic societies improved fermentations as a way to preserve their foods and beverages. Since then, human-associated *S. cerevisiae* yeasts have been exposed to selective pressures due to fluctuating stresses occurring during fermentations, such as osmotic stress, ethanol toxicity, anaerobic stress, acid stress, nutrient limitation, etc. (Querol et al., 2003). As a result of this passive domestication, human-associated *S. cerevisiae* yeasts exhibit differential adaptive traits and conform genetically separated populations (Gallone et al., 2016; Duan et al., 2018; Legras et al., 2018; Peter et al., 2018), according to their sources of isolation rather than to their geographic origins.

One of the most important selective pressures imposed to *S. cerevisiae* is ethanol tolerance. High ethanol concentrations also have a strong effect on *S. cerevisiae* yeast growth and metabolic efficiency (Ansanay-Galeote et al., 2001). Ethanol is a small amphipathic alcohol that can cross through cell membranes, increasing their fluidity and permeability, interfering in the folding and activity of proteins, and also affecting intracellular redox balance and pH homeostasis (reviewed in Auesukaree, 2017).

It is sometimes hard to differentiate between tolerance and resistance because they are defined in different ways depending on the research field. Most of the literature related to ethanol stress uses both concepts as synonymous to refer to the ability of yeasts to grow and survive in the presence of ethanol, although “ethanol tolerance” is the most frequently used (Snoek et al., 2016). In an attempt to differentiate these terms in microbiology, Brauner et al. (2016) defined resistance as the ability of a microorganism to grow in the presence of high concentrations of a drug, resulting in a higher minimal inhibitory concentration (MIC), and tolerance as the ability of the cell to survive the transient presence of a drug above the MIC. As ethanol is the main product of the *Saccharomyces* respire-fermentative metabolism, and, as mentioned, the basis of the “make-accumulate-consume” strategy, *Saccharomyces* yeasts acquired mechanisms to survive the transient presence of ethanol, and hence, we consider that the term “ethanol tolerance” would be mor appropriate.

Different studies have been devoted to understand the molecular mechanisms responsible of yeast response and tolerance to ethanol (for a review see Snoek et al., 2016). However, ethanol tolerance is a multilocus trait, not well characterized, because genes related to ethanol tolerance are broadly distributed throughout the genome (Giudici et al., 2005). In fact, as many different cellular processes are affected by ethanol, more than 200 genes have been linked to ethanol tolerance. Therefore, although many efforts have been made, mechanisms of ethanol tolerance are not fully understood yet.

In recent years, researchers have looked at adaptation to different stresses (Yona et al., 2012; Voordeckers et al., 2015; Adamczyk et al., 2016), including ethanol, in non-tolerant yeast exposed to gradually increasing stress levels. An interesting outcome of these experiments was the fixation in yeast of different genome rearrangements of adaptive value (Gorter de Vries et al., 2017).

In a previous study, we determined significant differences in ethanol tolerance between natural and fermentative *S. cerevisiae* strains, including strains isolated from different sources, from wine to traditional fermentations of Latin America (Arroyo-López et al., 2010). In the present study, we have sequenced the genomes of the most and least ethanol-tolerant *S. cerevisiae* strains reported in Arroyo-López et al. (2010) study to determine if they differ in their chromosomal constitution.

## Materials and Methods

### Strains and Sequencing

The *S. cerevisiae* strains used in this study are those exhibiting extreme differences in their ethanol tolerance (Arroyo-López et al., 2010). Temohaya-MI26 was isolated from the fermentation of Mezcal production in Durango, Mexico, and shows the lowest ethanol tolerance. Wine strain T73 was selected as a commercial dry yeast (Querol et al., 1992). It was isolated from a red wine fermentation in Alicante, Spain, and possesses an intermediate ethanol tolerance. Finally, strains CECT10094 and GBFlor-C are *flor* strains isolated from red Pitarra wine in Extremadura, Spain, and González Byass Sherry wine (Esteve-Zarzoso et al., 2001) in Jérez de la Frontera, Spain, respectively. They both exhibit the highest ethanol tolerances.

Yeast cells were grown in an overnight culture of GPY in 5 ml. Cells were pelleted in a microcentrifuge and suspended in 0.5 ml of 1 M sorbitol-0.1 M EDTA, pH 7.5. Then, they were transferred to a 1.5 ml microcentrifuge tube, with 0.02 ml of a solution of Zymolyase 60 (2.5 mg/ml). A microcentrifuge was used to spin down cells for 1 min, which were suspended in 0.5 ml of 50 mM Tris-HCl-20 mM EDTA, pH 7.4. After suspension, 0.05 ml of 10% sodium dodecyl sulfate was added and the mixture was incubated at 65°C for 30 min. Then, 0.2 ml of 5 M potassium acetate was added and the tubes were placed on ice for 30 min. Then they were centrifuged at maximum speed in a microcentrifuge for 5 min. Supernatant was transferred to a fresh microcentrifuge tube, and the DNA was precipitated by adding one volume of isopropanol. After incubation at room temperature for 5 min, the tubes were centrifuged for 10 min. The DNA was washed with 70% ethanol, vacuum dried, and dissolved in 50 μl of TE (10 mM Tris-HCl, 1 mM EDTA, pH 7.5). T73 was sequenced with a 300-bp paired-end library in an Illumina HiSeq 2500 equipment. Strains CECT10094, GBFlor-C and Temohaya-MI26 were sequenced with paired-end libraries of 100 bp with a mean insert size of 300 bp in an Illumina HiSeq 2500 instrument.

EC1118 sequencing data was downloaded from NCBI with identifiers: SRA, ERS484054; BioSample SAMEA2610549.

*S. cerevisiae* 2-200-2 is a diploid strain with a chromosome III trisomy used in the chromosome III removal experiment. This strain was obtained by Voordeckers et al. (2015) after evolving the haploid S288c-derivative strain FY5 in the presence of increasing levels of ethanol.

### Genomes Assembly and Annotation

Reads were trimmed with Sickle v1.2 (Joshi and Fass, 2011) with a minimum quality value per base of 28 at both ends and filtered at a minimum read length of 85 bp. A first preassembly step was carried out with Velvet v1.2.03 (Zerbino and Birney, 2008) to determine the best k-mer value for each library. The assembly was done with Sopra v1.4.6 (Dayarian et al., 2010) integrated with Velvet with the k-mer value determined in the previous step. Then, refinement of the results was carried out with SSPACE v2.0 (Boetzer et al., 2011) and GapFiller v1.11 (Boetzer and Pirovano, 2012) to improve scaffold length and remove internal gaps. Several rounds of Sopra/SSPACE/GapFiller were performed until the number of scaffolds could not be reduced. At each step of the process, the scaffolds were aligned against the reference genome of *S. cerevisiae* S288C with Mauve v2.3.1 (Darling et al., 2010). These steps can lead to overfitting and the nature of our sequence data mean we cannot verify any new recombination events, so, they were manually corrected. The final scaffolds were then aligned against the S288C reference genome with MUMmer v3.07 (Kurtz et al., 2004) and ordered into chromosomes with an in-house script.

Genomes annotation was done using two different strategies. First, the annotation from S288C genome was used to transfer to the new genomes by sequence homology using RATT (Otto et al., 2011). Second, an *ab initio* gene prediction was performed using Augustus web server (Stanke and Morgenstern, 2005), to complete the annotation of low homology regions where RATT was not able to transfer annotation. Both results were merged and the annotation was then manually corrected using Artemis (Rutherford et al., 2000) to remove false gene discovery and incorrect RATT transfer were either removed or corrected dependent on the nature of the mistake (e.g., wrong placement, lack of intron, etc.).

### Variants Detection and Chromosome Copy Number Analysis

Mappings against the reference *S. cerevisiae* S288C genome (version R64-2-1) were done using bowtie2 v2.3.0 (Langmead and Salzberg, 2012) with default parameters. Read Depth (RD) for each position was computed with bedtools v2.17.0 (Hung and Weng, 2017). To smooth the representation of RD by chromosome, a sliding windows analysis was performed. Mean mapping reads was calculated for 10kb windows moving by 1,000 nt. Variant calling analysis was performed with breseq v0.27.1 (Barrick et al., 2014) pipeline with polymorphisms mode to enable heterozygotic variants to be called. Minimum polymorphism frequency was set to 0.15 to avoid low frequency variants calling. Variants annotation and manipulation was done with gdools v0.27.1 from breseq package. Variants whose frequency was higher than 0.95 were considered homozygotic and they were considered heterozygotic if it was lower. R and ggplot2 package were used for data representation.

### Phylogenetic Analysis

All gene sequences were extracted from the annotations of the genomes assembled, as well as sequences from 38 strains representatives of different known clades (Supplementary Table S1). Orthologous genes among *S. cerevisiae* strains were translated and aligned with MAFFT v7.221 (Katoh and Standley, 2013). Then the alignments were back translated to nucleotides, and concatenated. Maximum Likelihood phylogeny was performed on the concatenated genes alignment with RAxML v8.1.24 (Stamatakis, 2014) with model GTR-Γ and 100 bootstrap replicates. The concatenated-gene ML tree was drawn with R and ggtree package (Yu et al., 2017).

### Determination of the Ploidy by Flow Cytometry

The total DNA content of the strain of interest was estimated by flow cytometry analysis in a BD FACSVerse cytometer following the SYTOX Green method as described in Haase and Reed (2002). Ploidy levels were scored on the basis of fluorescence intensity compared to the reference haploid S288c and diploid FY1679 *S. cerevisiae* strains. The estimated ploidy of the strains was obtained from three independent measurements.

### Expression Analysis

The expression data from a previous work on ethanol response (Navarro-Tapia et al., 2016) was used in this study (GEO accession: GSE44863). In brief, transcriptomic analysis come from a microarray analysis after ethanol shock. Temohaya-MI26 and CECT10094 were subjected to a 10% ethanol treatment and RNA was extracted 1 and 10 h later. As a control, RNA was extracted after 1 and 10 h of growth without ethanol treatment. Samples were hybridized for each condition against a pool of all the samples from all the conditions in the analysis. Expression data is reported as the log2 of the ratio of signal intensities between each condition and the pool. After combining each replicate, the genes were assigned to chromosomes according to their systematic names. Wilcoxon-Test implemented in ggpubr package v0.1.7 was used for the statistical analysis of differences between the expression of the chromosomes.

### Aneuploidy Analysis in *S. cerevisiae* Strains

Ploidies, aneuploidy presence, and 15% ethanol tolerances were extracted from the recent study of 1011 *S. cerevisiae* genomes (Peter et al., 2018). The 1011 strains were grouped by ploidy level and the presence of chromosome III copy number variations. Wilcoxon paired test was used to test differences between euploid diploids and the rest of ploidies and aneuploidies. Relative growth rates are represented as the normalization of the ratio between growth on standard YPD at 30°C and the stress condition (Peter et al., 2018).

### Yeast Chromosome III Removal

A counter selectable marker (Kutyna et al., 2014) was used to remove a single copy of chromosome III in *S. cerevisiae* 2-200-2 strain (Voordeckers et al., 2015) to obtain a derivative strain with one less copy of chromosome III, named as 2-200-2-S4. An integrative cassette targeted to a wide intergenic region (YCR027C-YCR028C) was synthetized from pCORE5 vector using the following primers: CHRIIIdel_F: CTGTAGCCATATTAAATTCCTTTGTCTCTGGACTCTTTCGAGCCCCCGATTTAGAGCTT and CHRIIIdel_R: TTAACGTTCAAGCAGCGTCAGTGAGAACTAAAATCATCCAATCTCGAGGTCGACGGTATCGAT. The 2-200-2 strain was transformed and colonies were selected in GPY with G418. Correct integration was corroborated by PCR using the following primers: test-CHRIII del_F: TCGACATCATCTGCCCAGAT and test-CHRIII del_R: ACTTAGGTGGAGGAGCAAG. After overnight growth in GPY (2% glucose, 0.5%, peptone, 0.5% yeast extract), cells were plated on galactose counter selection media (2% galactose, 0.5% peptone, 0.5% yeast extract), and colonies were used to measure chromosome III copy numbers.

### Chromosome III Copy Number Measurements

Genomic DNA was isolated and ethanol precipitated from the GPY liquid cell suspension in five independent culture replicates of 2-200-2 and 2-200-2-S4. DNA purity and concentration were determined in a NanoDrop ND-1000 spectrophotometer (Thermo-Scientific), and the integrity of all samples was checked by electrophoresis in agarose gel (1%). The PCR primers used to study the chromosome III copy number were designed from the available genomic sequence of *S. cerevisiae* strain S288C (R64-2-1, *Saccharomyces* genome database^1^). The sequences of PCR primer pairs used in this study are: ARE1-F: CCTCGTGTACCAGATCAAC; ARE1-R: AGGAAGATGGTGCCAATGAT; YCL001W-A-F: TGCTACGGTGGTTCTGCAAG; YCL001W-A-R: ACCACTGTGTCATCCGTTCT; POF1-F: TAATGGAGAGCTTCATGTCGGG; POF1-R: CCCTCAAGGATGTCACTGGC; ACT1_F: ATGTTCCCAGGTATTGCCG; ACT1_R: GCCAAAGCGGTGATTTCCT; YFR057W-F: ACACCGCCAAGCTTCCAATA; YFR057W-R: TTGCCACGCAAAGAAAGGAC; ACT1_F: CATGTTCCCAGGTATTGCCG; ACT1_R: GCCAAAGCGGTGATTTCCT; YFR057W-F: ACACCGCCAAGCTTCCAATA and YFR057W-R: TTGCCACGCAAAGAAAGGAC. Primers were designed to get amplicons of 100–200 bp in size to ensure maximal PCR efficiency, and the accuracy of quantification. PCR amplification was performed in a 10-μL final volume that contained 2.5 μL of the DNA template, 1.5 μL MilliQ water, 0.2 μM of each primer, and 5 μL of LightCycler 480 SYBR Green I Master (Roche). Reactions were performed in 96-well plates in an LightCycler 480 (II) PCR amplification and detection instrument with an initial denaturalization step at 95°C for 5 min, followed by 45 cycles of 95°C for 10 s, either 53 or 54°C for 10 s and 72°C for 4 s. A melting curve analysis was included at the end of each amplification program to confirm the presence of a single PCR product of all the samples with no primer-dimers. The Advanced Relative Quantification program v.1.5.1, implemented in the LightCycler 480, was used to analyze the results, and the efficiency of all the primer pairs was previously determined and included in the analysis. Normalization of the quantification results of genes *ARE1*, YCL001W-A, and *POF1* was performed using the levels of genes *ACT1* and *YFR057W* as reference genes.

### Ethanol Tolerance Assays by Drop Test Experiments

Drop test experiments were carried out to assess strains 2-200-2 and 2-200-2-S4 ethanol tolerances. Rectangular GPY plates supplemented with different ethanol percentages (0, 6, 10, 14, 16, and 18%) were prepared. Yeast cells were grown overnight at 28°C in GPY media and diluted to an OD_600_ = 0.1 in sterile water. Then, serial dilutions of cells (10^-1^ to 10^-3^) were transferred on the plates with replicates and incubated at 28°C for 10 days with the plates wrapped in plastic paraffin film to avoid ethanol evaporation. Each strain was inoculated twice in the same plate but in different positions, and an exact replicate of the plate was done. With this method, four biological replicates of each strain were generated.

## Results

### Phylogenetic Position of the Strains

**FIGURE 1:**
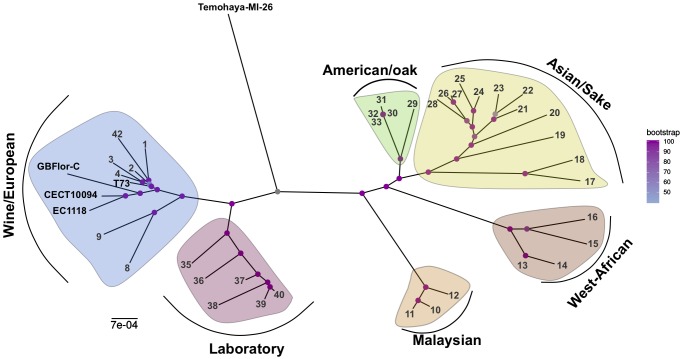
Phylogeny of *S. cerevisiae* strains from different origins. Multilocus Maximum Likelihood tree of 47 strains representative of different clades. Yellow: Asian/Sake, Green: American/Oak, Brown: West-African, Orange: Malaysian, Blue: Wine/European, Red: Laboratory. Strain references can be found in Supplementary Table S1.

We used whole genome sequencing of four strains, exhibiting different levels of ethanol tolerance (Arroyo-López et al., 2010), to investigate the relationship between genomic differences and ethanol tolerance. Our assembly and annotation pipeline allowed us to extract about 6,000 coding sequences per genome, of which 2115 concatenated gene sequences in common with other 38 strains (Supplementary Table S1), representative of different pure lineages described for *S. cerevisiae* (Liti et al., 2009), were used to reconstruct a multi-locus ML phylogeny (Figure 1). When looking at the placement of our strains we observed that Temohaya-MI26 does not appear to cluster within any of the groups we selected, and shows a central position in the tree. This strain may be from a different American population not considered here, like the recently described Ecuadorean population (Peter et al., 2018). The wine strains (T73 and the two *flor* strains) clustered with wine/European strains, but within two sister-clades. More specifically, the *flor* strains GBFlor-C and CECT10094 group with EC1118 and T73 in the other clade which contains wine strains. The position of EC118 is consistent with previous results (Coi et al., 2017) describing that strain EC1118 clusters with *flor* strains which form a subpopulation among wine/European strains. As EC1118 was closely related to high ethanol-tolerant strains, and showed an intermediate tolerance, we used published genomic data of this strain for further analysis.

### Heterozygosity Levels Differ Between Strains

The frequency of sexual reproduction and outcrossing in *S. cerevisiae* has a high impact on heterozygosity levels, which can indicate differences in life-style between strains. We assessed heterozygosity levels here calculating the number of heterozygous positions in coding regions of the studied strains. High differences are found between strains (Supplementary Table S2). Temohaya-MI26 has the lowest heterozygosity with 2433 hetrozygous positions in the genome. T73 and GBFlor-C have 4586 and 3094 heterozygous positons, respectively. However, EC1118 and CECT10094 have the highest heterozygosity levels with 12983 and 13789 heterozygotic SNPs in the genome, respectively, which represent a mean of two SNPs per gene in their genomes. Interestingly, no relationship is observed between ethanol tolerance and differences in heterozygosity.

**FIGURE 2:**
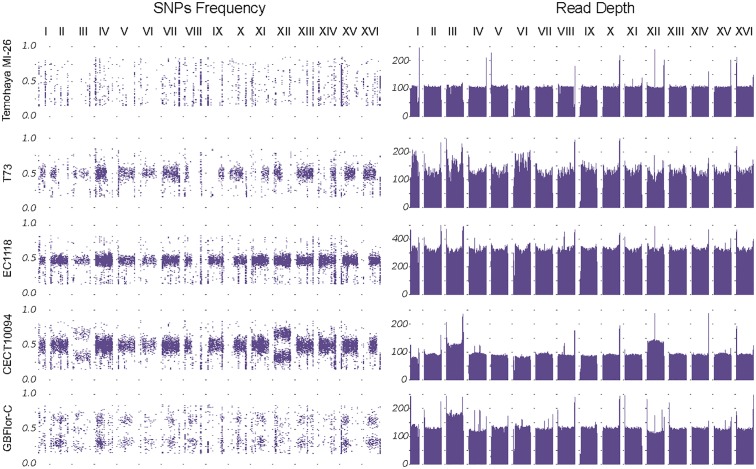
Genome structure of the strains. Left panel: SNP frequencies along the genome. Frequency distribution shows the different ploidies and aneuploidies present. Right panel: Read Depth by chromosomes, smoothed by the mean in 10 kb windows, confirm chromosome III aneuploidy in both GBFlor-C and CECT10094 and the chromosome XII trisomy in CECT10094.

In general, heterozygous SNPs were uniformly present along the genome, although several events of loss of heterozygosity (LOH) were observed (Figure 2 and Supplementary Figure S1). These events affected large chromosome portions, mostly including chromosome ends.

To identify possible genes involved in ethanol tolerance, we checked for non-synonymous SNPs fixed only in both highly ethanol-tolerant strains CECT10094 and GBFlor-C. Due to the heterozygosity and the phylogenic relatedness that these strains have with EC1118, only seven amino-acid changes were fixed and exclusive to both strains (Table 1). These are located in proteins encoded by six genes: *CUZ1, GCY1, RPN7, KAR3, DPB2*, and *ATG13*. With the exception of *CUZ1* and *GCY1*, these genes were located on the right arm of chromosome XVI, which was affected by a LOH event shared by CECT10094 and GBFlor-C. Interestingly, *CUZ1* and *RPN7* are two genes related with ubiquitin and proteasome pathways, which are important processes in the maintenance of protein homeostasis and the degradation of unfolded proteins. Both processes could be related with the presence of aneuploidies in the studied strains and their ethanol tolerance, as discussed below.

**Table 1:** Genes affected by non-synonymous changes exclusive of CECT10094 and GBFlor-C.

Systematic name	Gene name	Description	aa position	Ref aa	Alt aa
YNL155W	*CUZ1*	*CDC48*-associated UBL/Zn-finger protein	80	Y	F
YOR120W	*GCY1*	Galactose-inducible Crystallin-like Yeast prot	86	Q	E
YPR108W	*RPN7*	Regulatory Particle Non-ATPase	6	E	K
YPR141C	*KAR3*	KARyogamy	670	K	N
YPR175W	*DPB2*	DNA Polymerase B subunit 2	565	V	F
			584	E	Q
YPR185W	*ATG13*	AuTophaGy-related	158	T	S

### Highly Ethanol-Tolerant Strains Share Chromosome III Aneuploidy

Most strains of *S. cerevisiae* are diploids, but it has been shown that industrial strains, associated with human-related environments, present different ploidy levels (Gallone et al., 2016; Peter et al., 2018). We assessed our strains’ ploidy by flow cytometry, and found that T73, CECT10094, EC1118, and Temohaya-MI26 were diploids. Cytometry average of triplicates compared to a known diploid were respectively: 2.117 ± 0.029, 2.200 ± 0.030, 2.196 ± 0.029, 2.218 ± 0.027 (Supplementary Table S3). Contrastingly, GBFlor-C was found to be a triploid strain (3.510 ± 0.055) (Supplementary Table S3).

Another method to confirm the ploidy state is to use the heterozygotic SNP frequency distribution along the genome (Figure 2, left panel). The diploid and heterozygotic strains showed a SNP frequency distribution around 0.5, which confirms their diploid state. In the same way, GBFlor-C, which is triploid, showed a typical SNP frequency distribution around 0.33 and 0.66. As Temohaya-MI26 is completely homozygous, it was not possible to confirm its ploidy state with this method.

Fast adaptation to a stressful environment can be driven by large-scale genomic rearrangements. Among these, aneuploidies are getting much attention as a potential driver of adaptation of industrial relevance in *S. cerevisiae* (Gorter de Vries et al., 2017). We checked for the presence of aneuploidies in two ways: changes in read-depth between chromosomes and changes in heterozygous SNPs frequency compared to the overall genome frequency distribution (Figure 2). Interestingly, the highest ethanol-tolerant strains, CECT10094 and GBFlor-C were aneuploids. CECT10094 had an extra copy of chromosomes XII and III, and GBFlor-C also showed an extra copy of chromosome III. As chromosome III polisomy was shared between these two strains in different ploidy backgrounds, we further investigated if this could be of importance to explain their higher ethanol tolerance.

### Chromosome III Expression Increases With Ethanol Stress

**FIGURE 3:**
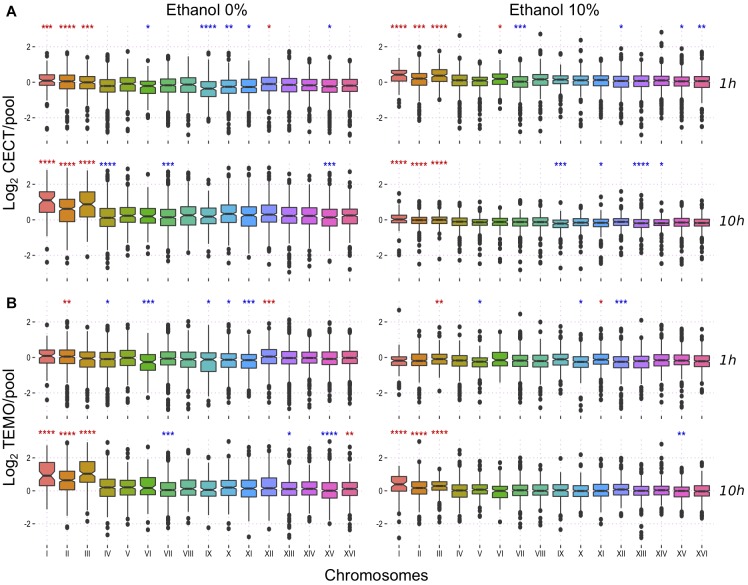
Transcriptional response to ethanol at a chromosomal level. **(A)** expression fold change of CECT10094 compared to the pool at 1 and 10 h growth with 0 and 10% ethanol stress. **(B)** expression fold change for Temohaya-MI26. Statistics Wilcoxon test among all groups: ns: *p* > 0.05, ^∗^*p* < 0.05, ^∗∗^*p* < 0.01, ^∗∗∗^*p* < 0.001, ^∗∗∗∗^*p* < 0.0001. Significance symbols are colored in red if the chromosome is upregulated and in blue if it is downregulated.

A higher number of copies of a chromosome is related with a higher expression of the genes in this chromosome (Torres et al., 2007). We asked if in this case the higher expression of chromosome III could be related with ethanol tolerance. We used transcriptomic data from a previous study (Navarro-Tapia et al., 2016) to shed light on the importance of chromosomes expression on the ethanol tolerance. In brief, the strain with the lowest and highest ethanol tolerance of a set of strains from diverse isolation sources (Temohaya-MI26 and CECT10094, respectively) were selected and their RNA was extracted after 1 or 10 h of growth in two conditions: after a 10% ethanol shock or without stress. The genes were grouped by chromosomes to show the global contribution of these in the expression profile of the different conditions (Figure 3). Contribution of each chromosome in the complete transcriptome of each strains were different but here we focused on chromosome III due to its aneuploidy in the highly ethanol-tolerant strains. Without ethanol stress, chromosome III showed a significantly higher expression at 1 h of growth compared to other chromosomes in CECT10094 but not in Temohaya-MI26. At 10 h of growth, chromosome III global expression is up-regulated in both strains and in both growth conditions. One hour after ethanol stress, however, chromosome III is significantly up-regulated in both Temohaya-MI26 and CECT10094. In Temohaya-MI26, chromosome III is the most significantly overexpressed chromosome in the genome after a short exposure to ethanol. Thus, the expression pattern observed here could be related with a higher expression in CECT10094 due to the aneuploidy. Furthermore, the change in the expression contribution of chromosome III in Temohaya-MI26 after ethanol shock is consistent with the presence of several genes in the chromosome contributing to the ethanol stress response, even in the low ethanol-tolerant strain.

### Chromosome III Aneuploidies Affect Growth on Ethanol in Different Backgrounds

**FIGURE 4:**
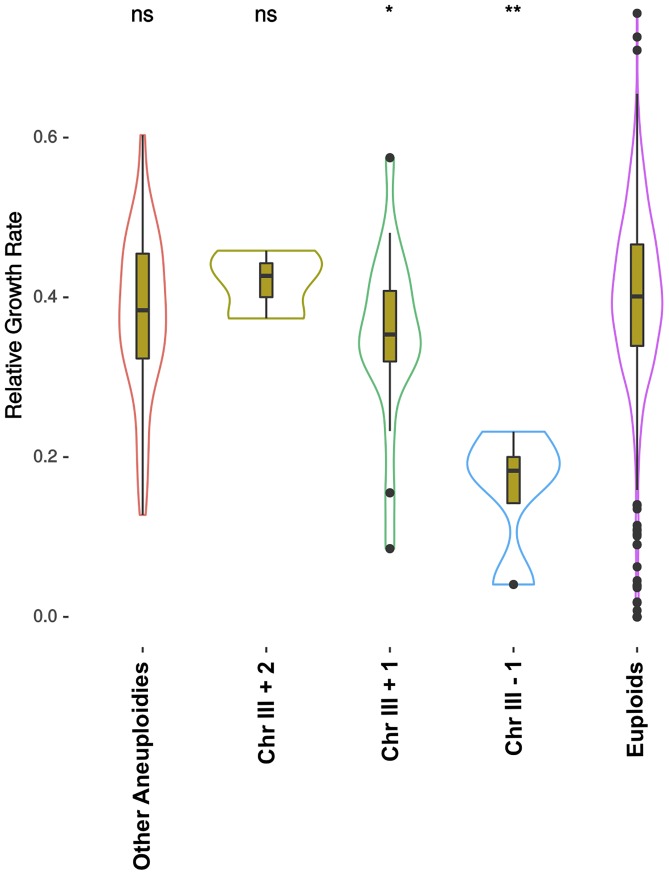
Relative growth rate of 1011 strains on 15% ethanol. Relative growth rate is the ratio between growth of the strain on YPD at 30°C and growth on 15% ethanol. Strains are grouped by their ploidy and presence of different aneuploidies. In this figure only diploids are presented, all other ploidies are shown in Supplementary Figure 2. Chr III +1, Chr III +2, and Chr III-1 tagged contain one extra copy, two extra copies and one less copy of chromosome III, respectively, and can exhibit other aneuploidies. “Other Aneuploidies” group are strains that have different kind of aneuploidies but not on chromosome III, and “Euploids” have no aneuploidy. Significance is Wilcoxon paired with diploid euploid strains as reference (ns: *p* > 0.05, ^∗^*p* < 0.05, ^∗∗^*p* < 0.01, ^∗∗∗^*p* < 0.001, ^∗∗∗∗^*p* < 0.0001). Original data were obtained from Peter et al. (2018).

Several studies showed that aneuploidies could be of importance in certain conditions (Gorter de Vries et al., 2017). In particular, chromosome III copy number variation was related with higher heat tolerance (Yona et al., 2012) and was duplicated in ethanol adaptation experiments (Voordeckers et al., 2015). In a recent study (Peter et al., 2018), more than 1000 *S. cerevisiae* strains were sequenced and phenotyped in several conditions. Here we used the phenotype on 15% ethanol stress and the ploidy and aneuploidy information, and grouped the strains to search for differences in ethanol stress tolerance in a wider genetic background set (Figure 4 and Supplementary Figure S2). Most of the ploidies and chromosome copy numbers did not show many differences compared to diploid strains exhibiting a perfect euploidy. As groups are of different sizes and many factors are involved in ethanol tolerance, differences are hard to see. However, in diploids the number of chromosome III copies showed a specific trend (Figure 4). Strains lacking one of the chromosome copy were significantly worse than diploids growing on 15% ethanol respect to control condition. As the number of copies of chromosome III increases, higher is the relative growth rate exhibited. Strains with an extra copy were better than the euploids with two copies, and these better than monosomic strains, with one single chromosome III.

### Removing the Extra Copy of Chromosome III Strongly Affects Ethanol Tolerance

To confirm that the aneuploidy on chromosome III directly influenced the ethanol tolerance of the strains, we removed the extra copy from the genome (see section Materials and Methods), returning strains to the euploid state. Unfortunately, we could not obtain any modified strain of CECT10094 and GBFlor-C with the experimental approach used. We therefore used a laboratory evolved strain (2-200-2) obtained by Voordeckers et al. (2015). These authors evolved six prototrophic, isogenic *S. cerevisiae* strains of different ploidy (1n, 2n, and 4n), all them generated from the haploid S288C-derivative FY5 strains, in chemostats with increasing ethanol concentrations (up to 12%) during 200 generations. The haploid and tetraploid lines showed rapid convergence toward a diploid state, and at the end of the experiment, all the evolved clones were highly tolerant to ethanol, and most of them shared the acquisition of an extra copy of chromosome III, although other specific aneuploidies were also present in some clones. The evolved clone 2-200-2 is a haploid-derived diploid with a chromosome III trisomy. As this evolved lab clone shared this genomic feature with our *flor* strains, it was used in an experiment to test if its ethanol tolerance was reverted after the removal of its extra chromosome III copy, by using the integration of a selection/counter-selection marker.

We first determined the copy number of chromosome III in the strain 2-200-2 and the modified strain 2-200-2-S4 by qPCR to confirm the removal of the extra copy. We used primers for three genes spread along the chromosome and compared chromosome dosage using as reference genes *ACT1* and YFR057W from chromosome VI. The results showed that 2-200-2-S4 had lost an extra copy of chromosome III for each one of the tested genes resulting in a gene copy number close to 2 (1.99 ± 0.31 for *ARE1*, 2.14 ± 0.28 for *YCL001W-A* and 2.06 ± 0.20 for *POF1*).

**FIGURE 5:**
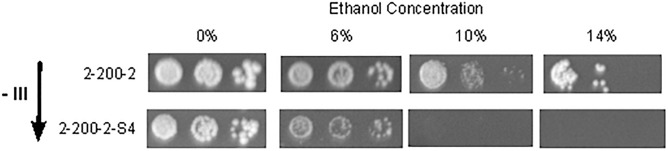
Removing the aneuploidy on chromosome III decreases ethanol tolerance. Drop test assays of strains 2-200-2 and 2-200-2-S4 in ethanol plates. Plates were incubated 10 days at 28°C. 2-200-2 had three copies of chromosome III and 2-200-2-S4 had two copies. Removing the aneuploidy affects ethanol tolerance.

After removing chromosome III extra copy, we tested growth of strains 2-200-2 and 2-200-2-S4 on GPY with different ethanol concentrations (Figure 5). The strain 2-200-2, which has three copies of chromosome III, was able to grow even on 14% ethanol concentration. 2-200-2-S4, which had the extra copy of the chromosome III removed, was not able to grow on 10%-ethanol medium.

## Discussion

Ethanol is one of the major stresses suffered by yeasts in industrial environments. Among the different species of its genus, *S. cerevisiae* is the most tolerant to ethanol (Arroyo-López et al., 2010). Even if this characteristic is widely studied due to its importance in biotechnology and industry, it is still unknown what are the key factors that drive adaptation to high ethanol concentrations (Snoek et al., 2016). Here, we sequenced the genome of *S. cerevisiae* strains especially selected for their differential ethanol tolerance. A previous work showed that Temohaya-MI26 was low ethanol-tolerant, T73 and EC1118 had an intermediate tolerance and GBFlor-C and CECT10094 were high ethanol-tolerant (Arroyo-López et al., 2010).

The phylogenetic analysis performed showed that the wine strains could be divided in two subclades. The first one grouped typical wine strains and contained the T73 strain, and the second grouped *flor* strains (GBFlor-C and CECT10094) with EC1118. These results confirm that *flor* strains form a different subpopulation among wine strains, as previously described (Legras et al., 2014, 2016; Coi et al., 2017; Eldarov et al., 2018). Temohaya-MI26, in contrast, was not included in any of the groups considered. The ethanol tolerance was higher in *flor* strains but not in EC1118 which is in the same group and closely related to CECT10094 and GBFlor-C. This points that this phenotype is variable even within the same population.

Until recently, most of the sequenced *S. cerervisiae* strains were homoploid spore derivatives to improve assembly and analysis. These methods nevertheless shadow interesting parts of genome structure. Heterozygosity levels were related to differences in strains lifestyle (Magwene et al., 2011). In industrial environments, this species reproduces asexually and has higher heterozygosity levels than natural strains (Gallone et al., 2016; Peter et al., 2018). The strains studied also showed similar trend. Temohaya-MI26, which is not related to industrial strains, showed a low heterozygosity. This may mainly be due to the use of haploselfing in its environment. In contrast, wine related strains showed higher heterozygosity, with events of LOH, and were in the range of levels previously described for wine strains (Gallone et al., 2016; Peter et al., 2018).

We found that the highly ethanol-tolerant strains shared an aneuploidy on chromosome III in different ploidy backgrounds. A high fidelity of genome replication and segregation is vital for the survival of any organism as well as for the production of future generations. Errors in these steps during meiosis, and also during mitosis in unicellular organisms, can lead to a change in ploidy or chromosome numbers. In fact, it has been suggested that the ethanol itself could induce chromosome malsegregation (Crebelli et al., 1989). These severe genome changes can be detrimental, causing a decrease in the fitness of the organism. However, during specific circumstances, such as periods of stress, in which gene dose increase can be beneficial, polyploidy or aneuploidy can provide a higher fitness (Todd et al., 2017). Aneuploidy is gaining attention for its relevance in industrial *S. cerevisiae* strains (Gorter de Vries et al., 2017) and for its possible implications in driving adaptation in general (Chen et al., 2012; Bennett et al., 2014). Consequences of aneuploidy are usually detrimental for strain growth (Mangado et al., 2018). However, it was described that specific chromosome copy-number variations could improve resistance to specific stresses. This way, chromosome III aneuploidy was related with improvement of heat tolerance (Yona et al., 2012). Other authors also found that chromosome III aneuploidies were generated as a response to ethanol stress (Gorter de Vries et al., 2017). Moreover, artificial segmental aneuploidies of chromosome III increased ethanol tolerance (Natesuntorn et al., 2015). Evolution on mild ethanol stress showed that different aneuploidies appeared, including chromosome III copy number increases (Adamczyk et al., 2016). Finally, a long-term evolution study showed that chromosome III aneuploidy was a common event (Voordeckers et al., 2015). Here, we found that relationship in non-laboratory strains, which may indicate that it is a long-term adaptation and its fixation seems to be important for ethanol tolerance. We also showed that the number of copies of the chromosome plays a role in this phenotype in different backgrounds (Figure 4), and that it is adaptive and affects directly to ethanol tolerance.

We dissected the expression profile by chromosomes of the high and low ethanol-tolerant strains. We found that the low ethanol-tolerant strains up-regulated chromosome III expression after ethanol stress and that the high ethanol-tolerant had its expression increased even in the absence of ethanol. Therefore, aneuploidy can be a way to change dosage of important genes present in chromosome III (Yona et al., 2012). This is consistent with our results, but further investigation is needed to find which genes in this chromosome could be involved in this process. Nevertheless, genes present in aneuploid chromosomes can change expression of other genes in other chromosomes, causing broad expression changes (Selmecki et al., 2008).

Aneuploidy itself affects the cell in different ways. Additional copies of a chromosome increases proteotoxic stress, which affect the protein folding processes in the cell (Torres et al., 2007). Interestingly, yeast were found to induce unfolded protein response under ethanol stress (Navarro-Tapia et al., 2016, 2017). Two out of seven nonsynonymous changes found in the high ethanol-tolerant strains affected genes related to protein homeostasis. We open here the possibility that ethanol and aneuploidy tolerance could involve similar processes, which may involve fixing variants affecting these processes and therefore aneuploidy itself could play a role on improving ethanol tolerance. As chromosome III is one of the smallest chromosome in *S. cerevisiae* genome, we cannot discard that the aneuploidy tolerance induced could be the cause of the observed phenotype.

## Conclusion

In conclusion, in this work we showed that ethanol tolerance was related to an aneuploidy on chromosome III in wine *S. cerevisiae*. Further work will be needed to elucidate the actual mechanism by which this phenomenon happens, but we confirmed that this is an adaptive trait that seems to be a widespread trend.

## Data Availability

All the genomic data in this article are available on NCBI under BioProject PRJNA493718.

## Author Contributions

EB and CT conceived and designed the study. MM and LM performed all the genome sequence and phylogenetic analyses under CT and EB supervision. AA and RP-T did the chromosome III removal. ML-P carried out the ethanol tolerance assays by drop tests. MM, RP-T, and CT wrote the first versions of the article, and EB the final version.

## Conflict of Interest Statement

The authors declare that the research was conducted in the absence of any commercial or financial relationships that could be construed as a potential conflict of interest.

## Footnotes

1 http://www.yeastgenome.org

## Supplementary Material

FIGURE S1 CIRCOS plot of heterozygous and homozygous SNPs. The wine and *flor* strains reads were mapped on T73 assembly for clarity purposes as described in Materials and Methods. Only SNP’s on coding sequences are represented. In blue are represented heterozygous SNP’s and in orange homozygous SNPs. Clear regions of LOH are observed on different chromosome regions.

FIGURE S2 Relative growth rate of 1011 strains on 15% ethanol. Relative growth rate on ethanol for all ploidies and aneuploidies (see Figure 4).

TABLE S1 Source of the *S. cerevisiae* genome sequences used in this study.

TABLE S2 Heterozygosity levels for each strain. The absolute number of heterozygotic and homozygotic SNPs in coding sequences are shown. The ratio is estimated as the number of heterozygotic positions divided by the total number of SNPs.

TABLE S3 Flow cytometry ploidy estimates.
