# Supplementary figures and images for: Aneuploidy and Ethanol Tolerance in Saccharomyces cerevisiae
Source: Front Genet. 2019 Feb 12;10:82. doi: 10.3389/fgene.2019.00082 (PMC6379819; doi:10.3389/fgene.2019.00082)

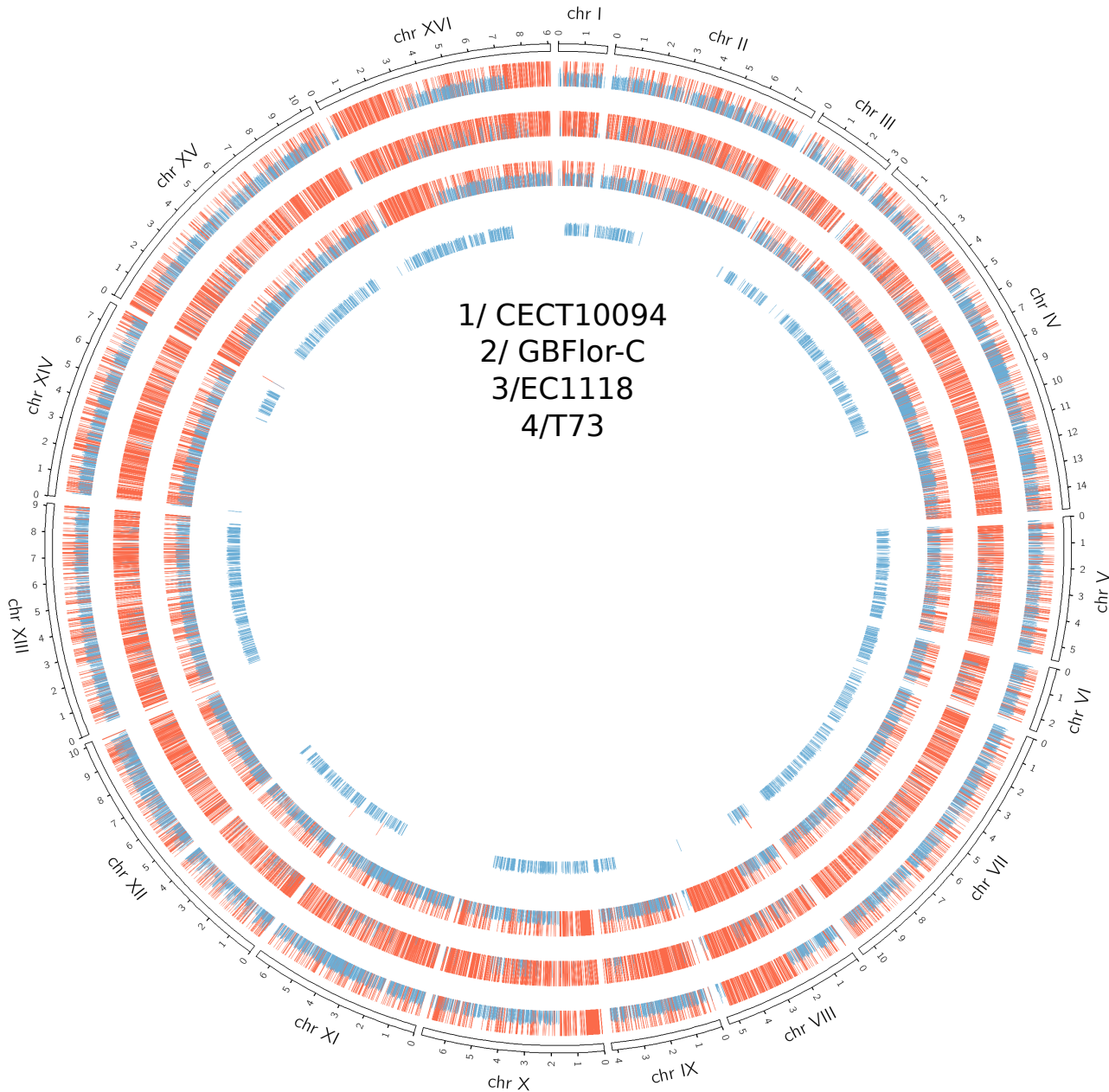

Supplement: FIGURE S1 — CIRCOS plot of heterozygous and homozygous SNPs. The wine and flor strains reads were mapped on T73 assembly for clarity purposes as described in Materials and Methods. Only SNP’s on coding sequences are represented. In blue are represented heterozygous SNP’s and in orange homozygous SNPs. Clear regions of LOH are observed on different chromosome regions. [file Image_1.pdf]

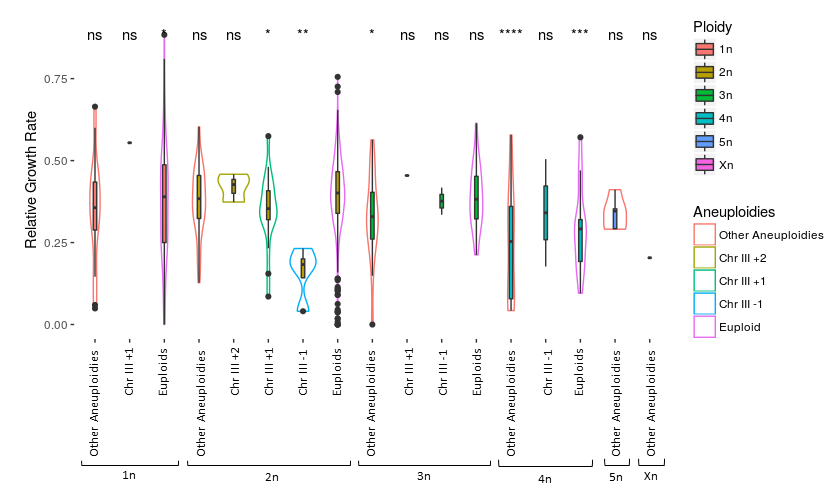

Supplement: FIGURE S2 — Relative growth rate of 1011 strains on 15% ethanol. Relative growth rate on ethanol for all ploidies and aneuploidies (see Figure 4). [file Image_2.tif]
